# A Rare Case of Cyclical Hemothorax: Thoracic Endometriosis Syndrome

**DOI:** 10.1155/2018/9830797

**Published:** 2018-08-19

**Authors:** Muhammad Shabbir Rawala, Muhammad Farhan Khaliq, Muhammad Asif Iqbal, S. Tahira Shah Naqvi, Kinaan Farhan, Andrew Myers, Kristen Helmick

**Affiliations:** ^1^Department of Medicine, Charleston Area Medical Center, Charleston, WV, USA; ^2^Department of Medicine, Jinnah Medical and Dental College, Karachi, Pakistan

## Abstract

Endometriosis is a common condition in which endometrial cells and stroma are deposited in extrauterine sites. Its prevalence has been estimated to be 10% of reproductive age females. It is commonly found in the pelvis; however, it may be found in the abdomen, thorax, brain, or skin. Thoracic involvement is a relatively rare presentation of this common disease. Thoracic endometriosis commonly presents as pneumothorax in 73% of patients. A rarer presentation of thoracic endometriosis is hemothorax (<14%) or hemoptysis (7%). Thoracic endometriosis is an uncommon cause of a pleural effusion. We present a case of 28-year-old African American female with no other medical conditions. She presented to the hospital with worsening right-sided pleuritic chest pain, dyspnea, and menorrhagia. She had been complaining of pleuritic chest pain for 5 years, the onset of which corresponds to the start of her menstrual cycle and is relieved with cessation of menses. Initial laboratory studies revealed a severe microcytic anemia with normal coagulation profile. Chest X-ray showed small right pleural effusion and suspicious for airspace disease. A computed tomography (CT) of chest was ordered for further clarification and identified large right pleural effusion. CT-guided thoracentesis removed 500 ml of serosanguinous fluid consisting of blood elements. There can be multiple sites involved with endometriosis and can present with wide range of symptoms that occur periodically with menses in young woman. The history and pleural fluid findings of this case are suggestive of Thoracic Endometriosis Syndrome. The diagnosis of this is often missed or delayed by clinicians, which can result in recurrent hospitalization and other complications. As internists we should be suspicious of atypical presentations of endometriosis and treat them early before complications develop. This case also highlights the importance of suspecting atypical etiologies for pleural effusion.

## 1. Introduction

Endometriosis is a common disorder that affects approximately 10% of the women in reproductive age and approximately 35–50% women with infertility [1, 2]. It is difficult to ascertain an accurate prevalence for endometriosis; it remains underdiagnosed as the diagnosis does require invasive testing.

Endometriosis is characterized by the presence of endometrial tissue, including the stroma and glands, located outside the uterine cavity [3]. Endometriosis most commonly occurs in pelvis, leading to symptoms like dyspareunia, dysmenorrhea, and dysuria. It can, however, involve other sites as well. Ectopic endometriosis has been found in genitals, abdomen, lungs, diaphragm, pleural cavity, and pericardium [4–8].

Thoracic endometriosis syndrome is an extremely rare disorder that involves presence of endometrial tissue in or around the lung but not limited to pleura, parenchyma, and the airways [9, 10].

Theories for pathogenesis of endometriosis are as follows.

Karl Von Rokitanski was the first one to identify endometriosis histologically under microscope [11].

There are three core theories that describe the pathogenesis of endometriosis:

1. Induction theory: it describes that endometriosis develops from metaplasia of cells lining the pelvic peritoneum [12]. Pleura and peritoneum both develop from coelomic epithelium; therefore, it cannot be excluded that diaphragmatic endometriosis originates from coelomic metaplasia.
2. Reflux theory: it explains that there is reflux of endometrial tissue through the fallopian tubes leading to implantation of endometrial tissue in other organs [13].
3. Metastasis theory: it basically describes the spread of endometrial tissue hematogenously through the uterine and pelvic vessels [14].

## 2. Case Report

We present a case of a 28-year-old African American female without any comorbid conditions who presented to the emergency department with right-sided pleuritic chest pain, dyspnea, and menorrhagia. She had been having intermittent pleuritic pain since 5 years and had been to the hospital many times in the past but without any diagnosis and resolution of her symptoms.

On examination, patient had stable vitals and her chest exam revealed absent breath sounds on right basal region.

Initial laboratory studies revealed a severe microcytic anemia with normal coagulation profile. Her initial chest X-ray showed right pleural effusion and airspace disease while computed tomography (CT) of chest identified large right pleural effusion.

She underwent ultrasound of the pelvis that revealed approximately 6 cm fibroid in uterine fundus.

**Figure 1:**
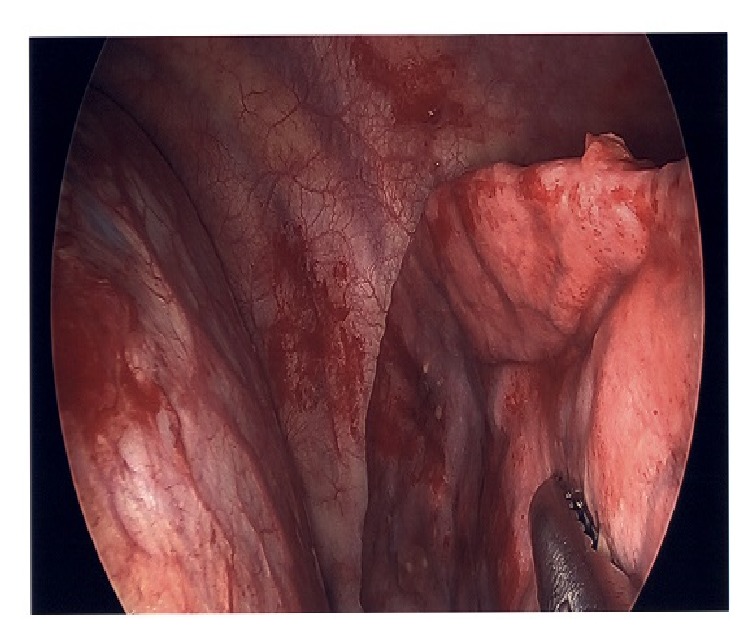
Images from VATS.

**Figure 2:**
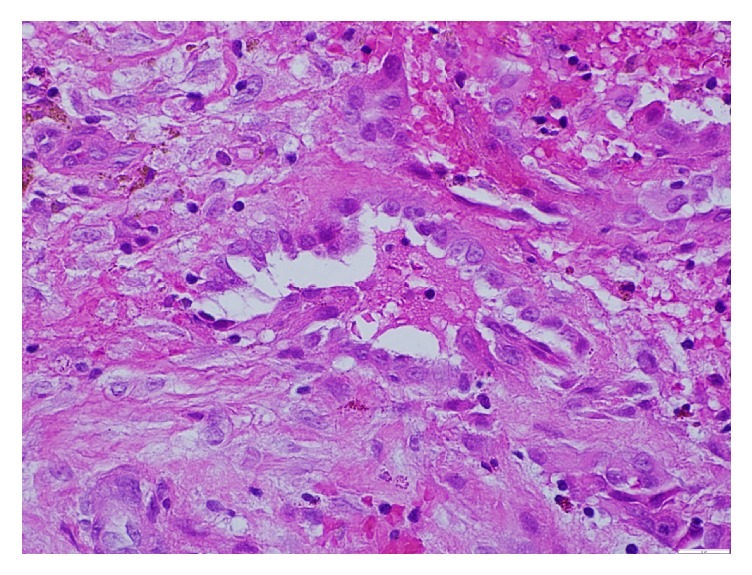
Epithelioid cells forming a structure reminiscent of glandular space.

**Figure 3:**
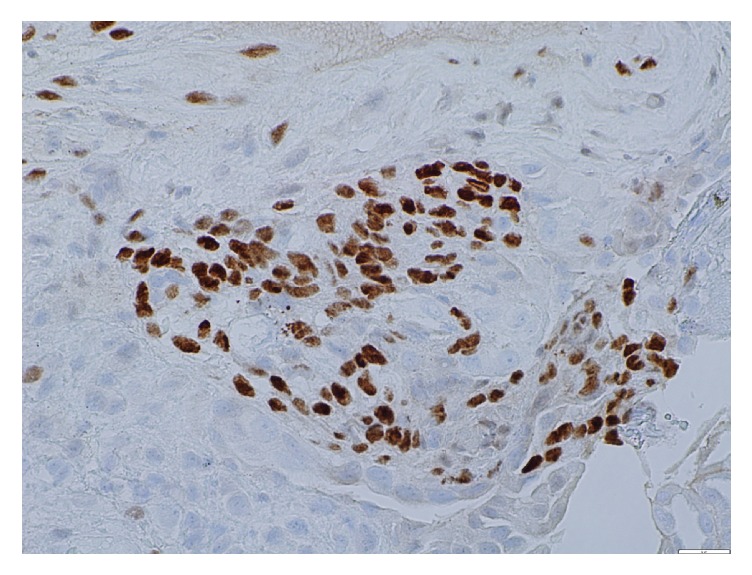
Glandular cells showing nuclear positivity with immunohistochemical staining.

Interventional Radiology was consulted for thoracentesis and 500 ml of serosanguinous fluid consisting of blood elements was drained. There was suspicion of thoracic endometriosis due to the temporal relationship between commencement of symptoms and menstrual cycle each month. The patient underwent video-assisted thoracoscopy surgery (VATS) that identified implants on diaphragm (Figure 1) and abnormal lung with remnants of hemorrhage in pleura. The specimens were studied histologically, and diagnosis of thoracic endometriosis was confirmed (Figures 2 and 3).

Patient was started on Leuprolide; however, after a few months, she stopped the treatment, as she was not able to tolerate it. She did have a relapse of her symptoms and again presented to emergency department where she was managed conservatively.

## 3. Discussion

Endometriosis affects an estimated 89 million women of reproductive age worldwide; in other words, it affects 6% to 10% of all women [1, 2, 15].

Prevalence of endometriosis being diagnosed in US women is estimated to be 6.1% [15]. The most common presenting symptoms are menstrual pelvic pain and/or cramping, nonmenstrual pelvic pain and/or cramping, and infertility and dyspareunia.

Endometriosis affects pelvic organs most frequently. Extra pelvic organs, less commonly, can also be affected. Thoracic and diaphragmatic involvement is a relatively rare presentation [9]. Thoracic endometriosis (TES) is the term implied to the presence of endometrial implants in airways, pleura, and lung parenchyma [9, 10].

Thoracic endometriosis is a rare condition, and diagnosis is often delayed. Amongst the women diagnosed with TES, 50-85% also have pelvic endometriosis. The percentage of women with pelvic disease who develop TES in their disease course is largely unknown [16]. The average age at presentation with TES is 35 years, with a range from 19 to 54 years [16, 17].

In 1938, Schwarz was the first author to characterize endometriosis of the lung parenchyma [18]. The mechanisms suggested for pathogenesis of TES are as follows: (1) tissue migration through pelvic vessels and (2) reflux of endometrial tissue through fallopian tubes into peritoneal cavity, then leading into thoracic cavity through diaphragmatic fenestrations/defects [19–22]. The thoracic diaphragm and visceral diaphragm are the most commonly described sites of lesions (38.8% and 29.6%, respectively), with the parenchyma less commonly reported [23, 24]. The distribution of endometrial implant through the diaphragm seems to be asymmetric with the right being affected more than the left; this can be explained by transportation of viable cells by the intra-abdominal current flowing in a clockwise manner coming down from the left peritoneal gutter and flowing across the pelvic floor and up along the right peritoneal gutter, once they reach the right upper quadrant, they are stuck by falciform ligament (peritoneal fold extending to the liver from diaphragm and abdominal wall). This phenomenon facilitates the seeding of endometrial implants to the right diaphragm and ultimately to thoracic cavity through fenestrations in diaphragm [25]. The diffusion of endometrial cells through fenestrations is evident in the literature due to impressive and almost identical preponderance of right-sided lesions of both diaphragm and pleura [13, 16]. This asymmetry in distribution argues against the theory of coelomic metaplasia; however due to embryonic origin of pleura and peritoneum from coelomic epithelium, this theory cannot be entirely rejected. We suggest that, in this particular case, endometrial implants on the parietal pleura itself may have been responsible for the associated pleural effusion.

Patients usually have catamenial symptoms, occurring within 24-48 hours of onset of menses. The most frequent symptoms the patient presents with are chest pain (90%), followed by dyspnea (31%), hemoptysis (7%), and cough (rare). Patients at initial workup are found to have pneumothorax (73%), hemothorax (14%), hemoptysis (7%), and pulmonary nodules (6%) [17].

Diagnostic methods for TES include chest X-ray (pleural effusion/opacities/nodular infiltrates), CT scan, or MRI (showing opacities, ground glass infiltrates, thin wall cavities, nodules, and bullous formation). These may be unremarkable if not performed during menses. Video-assisted thoracoscopy reveals diaphragm perforations and nodular or plaque like brown/violet deposits less than 1 cm in size), bronchoscopy may show some pin-like red submucosal lesions, and bronchial biopsy and brush cytology reveal endometrial cells [26–28]. Laparoscopy has been used to help in the investigations of patients presenting with isolated recurrent right-sided chest and upper abdominal pain [29].

There are multiple treatment options that include simple observation, surgical treatment, medical treatment, and combination therapy. Medical treatment consists of gonadotropin-releasing hormone analogues, aiming to suppress the hypophyseal-gonadal axis, ensuring a regression of the endometrial implants. If this fails then surgical resection of the endometriomas is suggested, although relapse rate may be high.

## 4. Conclusion

Our case does highlight the fact that endometriosis can be common in reproductive age women, but thoracic endometriosis is relatively rare and does require a high index of suspicion to diagnose. There can also be instances, like in our case, where the patient remained without a diagnosis for 5 years but was having symptoms regularly with each menstrual cycle.

It also teaches us to evaluate atypical etiologies of pleural effusion.
